# “Antimicrobial properties of tissue conditioner modified with chitosan and green-synthesized silver nanoparticles: a promising approach for preventing denture stomatitis”

**DOI:** 10.1186/s12903-024-03880-z

**Published:** 2024-01-31

**Authors:** Sina Safari, Mahmood Barani, Roya Sadrmohammadi

**Affiliations:** 1 Student Research Committee and Department of Prosthodontics, School of Dentistry, Kerman University of Medical Sciences, Kerman, Iran; 2 Medical Mycology and Bacteriology Research Center, Kerman University of Medical Sciences, Kerman, 7616913555 Iran; 3 Resident of Department of Prosthodontics, School of Dentistry, Kerman University of Medical Sciences, Kerman, Iran

**Keywords:** Chitosan, Silver nanoparticle, Candida albicans, Denture stomatitis, Green synthesis, Antifungal activity

## Abstract

**Background:**

Chitosan is known to inhibit the growth of many bacteria and fungi. Tissue conditioners are commonly used to prevent bone destruction under dentures. However, over time, these materials can become a suitable substrate for microbial growth. One approach to improving dental materials is the use of nanoparticles. This study examined the antifungal properties of chitosan and green technique-synthesized silver nanoparticles in combination with tissue conditioners.

**Methods:**

Tissue conditioner materials were mixed with chitosan and silver nanoparticles at concentrations of 0.097%, 0.19%, and 0.37%, along with 1.25 ppm Nystatin, and their antimicrobial properties against Candida albicans were investigated. The growth rate was measured after 24 h of incubation at 37 °C. Non-parametric tests, such as the Kruskal-Wallis H test and Mann-Whitney U test with Bonferroni correction, were used for data analysis after verifying that the groups did not have a normal distribution.

**Results:**

Compared with the control and Nystatin groups, the Chitosan-silver groups showed a significant decrease in the number of CFUs of Candida albicans.

**Conclusions:**

The combination of chitosan and silver nanoparticles with tissue conditioner materials is a promising alternative for preventing and treating denture stomatitis. These findings suggest that using very small amounts of nanoparticles in dental materials could effectively prevent microbial growth, which could improve the longevity and efficacy of dental prosthetics and materials.

**Supplementary Information:**

The online version contains supplementary material available at 10.1186/s12903-024-03880-z.

## Background

Tissue conditioners are used to treat and prepare the damaged tissues supporting dentures. these materials are used for palliative, and diagnostic purposes and to modify the occlusion and height of old prostheses [1]. Unfortunately, in some cases, tissue conditioners provide a suitable environment for the growth and colonization of all kinds of microorganisms, which can aggravate the complications caused by dentures [2].

One of the most common side effects of using complete dentures is denture stomatitis [3]. The etiology of denture stomatitis includes a set of factors. Candida has been recorded as one of the critical factors in the onset of this disease in people who use dentures [4]. Despite the rising incidence of candidiasis, current treatments primarily depend on azoles, polyenes, and echinocandins. These drugs frequently cause side effects, have drug-drug interactions, and exhibit limited effectiveness in certain tissues [5]. Furthermore, the emergence of fungal resistance, particularly against azoles, complicates treatment [6]. Additionally, the lack of antifungal vaccines [7] underscores the critical need to develop new, efficient, and safer antifungal agents to effectively target pathogenic Candida spp [7].

Combining antifungal and antimicrobial drugs with tissue conditioner materials can inhibit the formation of microbial plaque, especially candida biofilm, and effectively prevent and control denture stomatitis.

Silver ions are widely used as an antimicrobial agent against various bacterial strains and microorganisms. In recent years, the field of nanotechnology has developed new materials that have found success in a broad range of activities, from industrial processes to basic science [8]. Specifically, metal nanoparticles are a primary focus in nanomaterials, offering enhanced physical and chemical properties such as catalytic activity, stability, and optical and mechanical characteristics [9]. These materials have recently been utilized in antimicrobial formulations to combat pathogenic microorganisms. Notably, silver nanoparticles have demonstrated in vitro inhibitory effects on bacterial and fungal strains, using concentrations comparable to those of conventional antimicrobials [10]. Green synthesis is increasingly in demand due to its time-efficient approach and simplicity in laboratory conditions. Silver nanoparticles, recognized for their effective anti-inflammatory, antibacterial, and antifungal properties, are seeing growing use in the medical and dental fields. The green synthesis of these nanoparticles is proving to be an effective method, highlighting their importance in medicine and dentistry. This approach utilizes plant extracts, sugars, polymers, and microorganisms as reducing and capping agents in nano-synthesis. It offers the advantages of simplicity, easy reproducibility, and the production of stable by-products. Notably, green synthesis using plant extracts is relatively quicker in preparation time, yet much of this field remains unexplored to its full potential [11].

Additionally, functionalizing nanoparticles with biopolymers is emerging as a viable option to minimize adverse effects. Altering the surface chemistry of nanoparticles through functionalization enhances characteristics such as chemical stability, biological interaction with targets, and can facilitate the gradual release of the active ingredient [12]. Chitosan, a poly-cationic molecule produced by the chemical deacetylation of chitin, stands out among these polymers .Besides reducing the toxicity of nanoparticles, chitosan also exhibits antimicrobial properties against fungi and bacteria [13].

Chitosan affects the cell membrane through electrostatic interactions with negatively charged phospholipids. When the cell membrane is disrupted, chitosan can enter the cell. This can lead to the inhibition of DNA/RNA synthesis and disruption of protein synthesis [14, 15] The structure of chitosan features unique functional groups that are crucial for interactions with metal ions and nanoparticles. Specifically, the primary amino groups in chitosan bind with metal surfaces and serve as capping sites for nanoparticle stabilization [16]. As a result, chitosan can form composites with silver and other noble metals, whether in their ionic or metallic forms. This combination of nanoparticles and chitosan appears to be a promising candidate for biomedical applications, owing to its biodegradability, antibacterial efficacy, and exceptional chelating properties.

Due to the alarming spread of resistance to classical antimicrobial agents, innovative therapeutic methods to combat antibiotic-resistant pathogens such as plant-derived compounds and nanoparticles seem essential. This study investigated the antimicrobial effect of chitosan and biosynthesized silver nanoparticles using the Pistacia plant and compared it with Nystatin against attached C. albicans on tissue conditioners.

## Methods

### Preparation of silver nanoparticles by green technique

First, 2 g of the completely dried Pistacia plant (Collected from Kerman Province, Iran) were immersed in distilled water (100 ml) and placed on a heater stirrer for 25 min to boil. Then the solution was filtered using filter paper and the collected extract was stored in the refrigerator at 4 °C. Briefly, 20 mL of silver nitrate (Merck, Germany) solution was placed in an Erlenmeyer flask and placed in a paraffin bath with a temperature of 95 °C. Then 40 ml of aqueous solution was added to it. After removing the solvent, dark sediment was obtained.

### Preparation of chitosan silver nanocomposite

0.5 gr of chitosan (Merck, Germany) was dissolved in 1 ml of 1% acetic acid (Merck, Germany) and 49 ml of deionized water. 1 mM of Biosynthesized silver nanoparticles were added to the chitosan solution and stirred for 48 h. To separate the synthesized nanocomposite, the above solution was centrifuged for 15 min at 10,000 rpm(Sigma 1‑14) and dried at 60 °C for 24 h(MEMMERT universal cabinet UN30, 32 L) [17].

Silver nanoparticles give a specific vibrational peak in the region of 400 to 500 and 1500–1700^− 1^ cm in the IR spectrum, which was investigated by FTIR spectrometer (FTIR Thermo Avatar). Also, the morphology and size of the synthesized silver chitosan nanocomposite were investigated by SEM (FE-SEM TESCAN MIRA2).

### Preparation of tissue conditioner discs

Five types of tissue conditioner discs were prepared. A tissue conditioner without chitosan was used as a control. Chitosan and silver nanoparticles were ball milled for 4 h. For the experimental samples, part of the tissue conditioner powder was replaced with chitosan at concentrations equal to 1, 2, 4 times MIC, and 1.25 ppm of Nystatin, the antifungal gold standard and named in order into group 2, 3, and then the powder was mixed with the monomer according to the manufacturer’s recommendations.

**Fig. 1:**
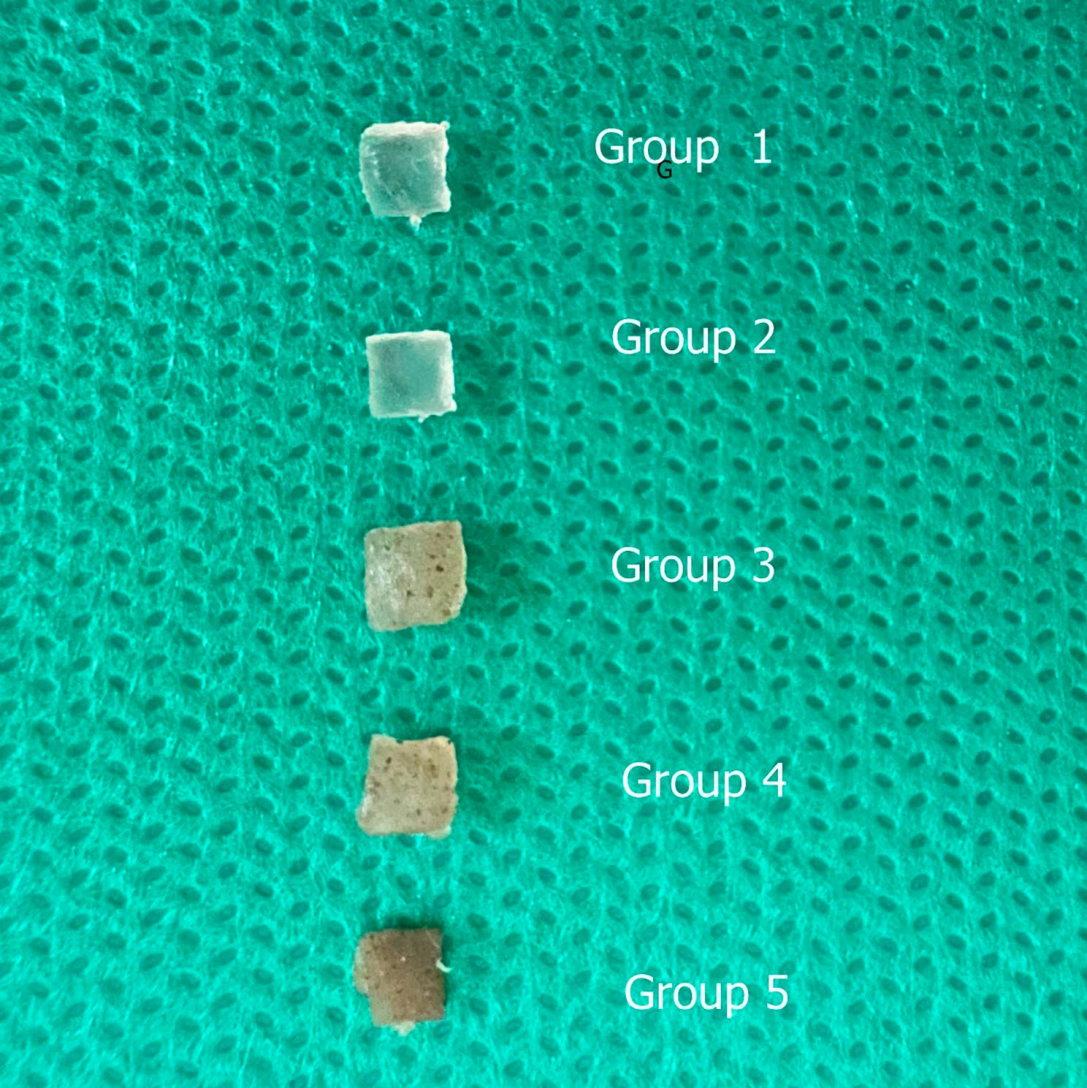
Five groups of prepared discs

The disks were classified into five groups: group one contained only tissue conditioner, group two contained tissue conditioner and nystatin, group three contained 1 MIC chitosan silver nanoparticle, group four contained 2 MIC chitosan silver nanoparticles, and group five contained 4 MIC chitosan silver nanoparticles.(Fig. 1).

The combined materials were poured into silicon molds and after setting, they were cut into 3 × 3 mm discs using a sterile scalpel (*n* = 6). The entire process was performed under aseptic conditions and all prepared discs were sterilized by UV for 15 min.

### Antifungal sensitivity test

This set includes two tests. At first, the minimum inhibitory concentration of chitosan silver nanoparticles was evaluated against C. albicans. Then, tissue conditioner discs were prepared and their effect on adherence of C. albicans was compared.

#### Determining the Minimum Inhibitory Concentration (MIC)

MIC was determined by the Broth Micro-Dilution method adapted from CLSI [18]. Stock solutions of chitosan and silver nanoparticles (2.5% by weight in volume) were prepared and the pH was adjusted between 5.6 and 5.8 [19].

Then, 50 µl of the stock solution was added to a sterile 96-well microplate, and two-fold serial dilutions were made. Then 50 µl of inoculum prepared from 1 × 10^5^ cells/ml Candida albicans was added to each well to obtain the final concentration of chitosan and silver nanoparticles from 25 to 0.048 mg/ml. The microplate was incubated in aerobic conditions at 37 ± 2˚ C for 24 h. The whole experiment was repeated three times.

The last well in which the change in appearance did not occur, the ninth well with a concentration of 0.097 mg/ml, was selected as MIC.

#### Investigation of antimicrobial effects

C. albicans was cultured on Sabouraud Dextrose Agar (SDA) (Merck, Germany). 1 ml of sterile Nutrient Broth (NB) was inoculated with a standard C. albicans colony. After 24 h, the inoculum size was adjusted to 0.5 McFarland units and the tissue conditioner discs were immersed in Eppendorf containing 900 µL of Sabouraud broth in triplicate. All discs were cultured with 100 µl of C. albicans suspension and incubated at 37 ± 2 °C for 24 h. After incubation, the broth was removed with a sterile pipette and the discs were washed five times with sterile water to loosen and detach the adherent cells.

Then the discs were placed in a sterile eppendorf containing 1 ml of sterile saline and placed in a vortex machine for 30 s to remove surface organisms. The discs were discarded and tenfold serial dilutions were made four times from the resulting liquid, and 100 µl of each eppendorf was seeded on SDA plates and incubated for 24 h at 37 °C. After incubation (Gallenkamp, England), the number of colony-forming units (CFU) was counted (Supplementary Material 1).

### Statistical analysis

Statistical analysis was performed using SPSS 26. Descriptive statistics, such as median and standard deviation, were used to describe the data. Non-normality of the groups was confirmed through the Kolmogorov-Smirnov test, and non-parametric tests, including the Kruskal-Wallis H test and Mann-Whitney U test with Bonferroni correction, were used for data analysis.

## Results

### FTIR test of chitosan silver nanocomposite

**Fig. 2:**
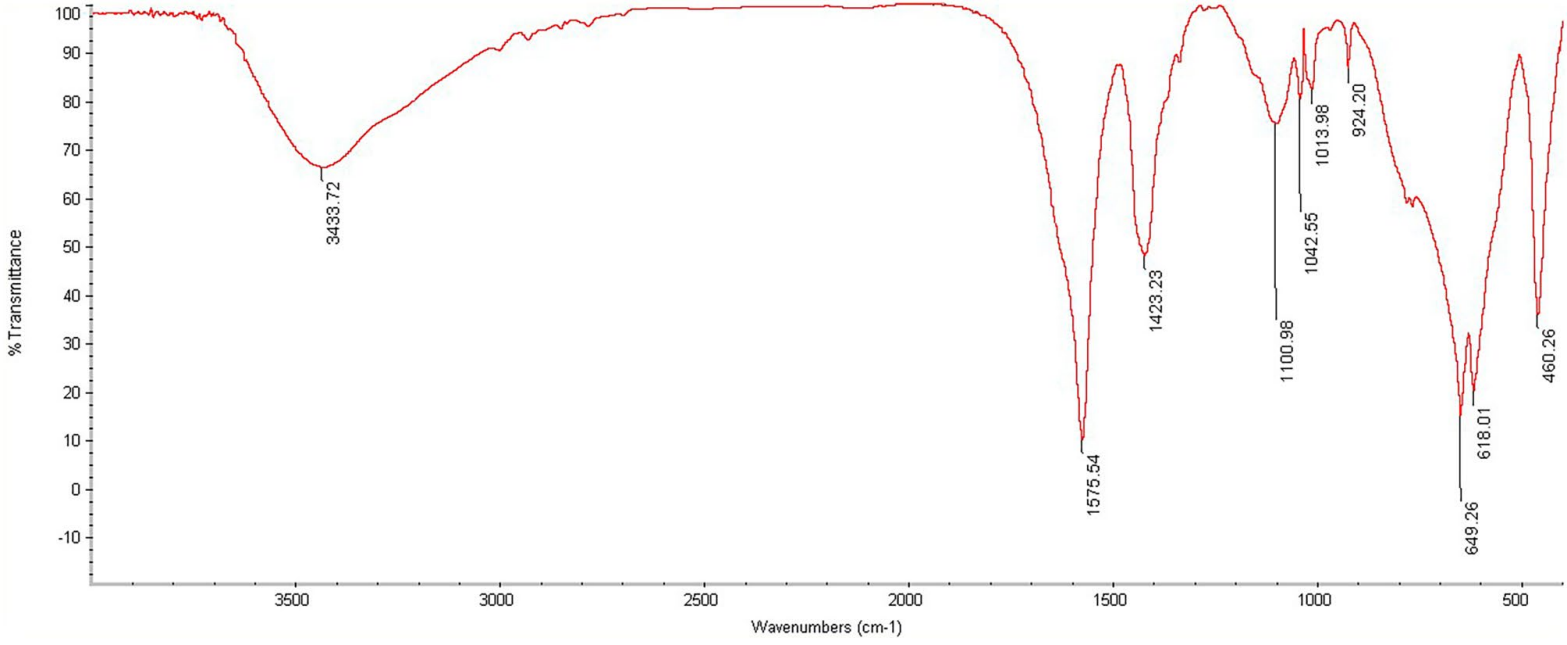
The FTIR spectrum of chitosan silver nanocomposite

The FTIR spectrum of chitosan silver nanocomposite is shown in Fig. 2. The characteristic band at 3433 cm^− 1^ is attributed to the frequency of N-H and OH stretching vibrations. The peaks at 1575, 1423, and 1100 cm^− 1^ are related to C = O stretching, N-H bending, and O-H bending vibrations, respectively. Also, the peak at 649 cm^− 1^ is related to Ag-O silver nanoparticles. In general, FTIR results indicate the successful synthesis of silver chitosan nanocomposite [20].

### Determining the morphology of chitosan silver nanocomposite with FESEM

**Fig. 3:**
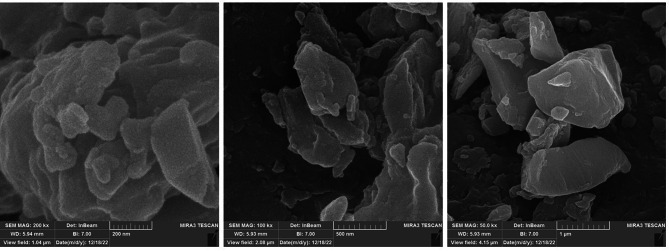
The morphology of chitosan silver nanocomposite

The morphology of chitosan silver nanocomposite is shown in Fig. 3. The nanocomposite has a nanometer and micrometer size distribution. Also, the morphology of the nanostructures shows that they have sharp and triangular edges. Also, the morphology of the nanostructures shows that they have sharp and triangular edges, which have been shown to have higher antimicrobial properties due to the destruction of the microbe’s membrane.

**Fig. 4:**
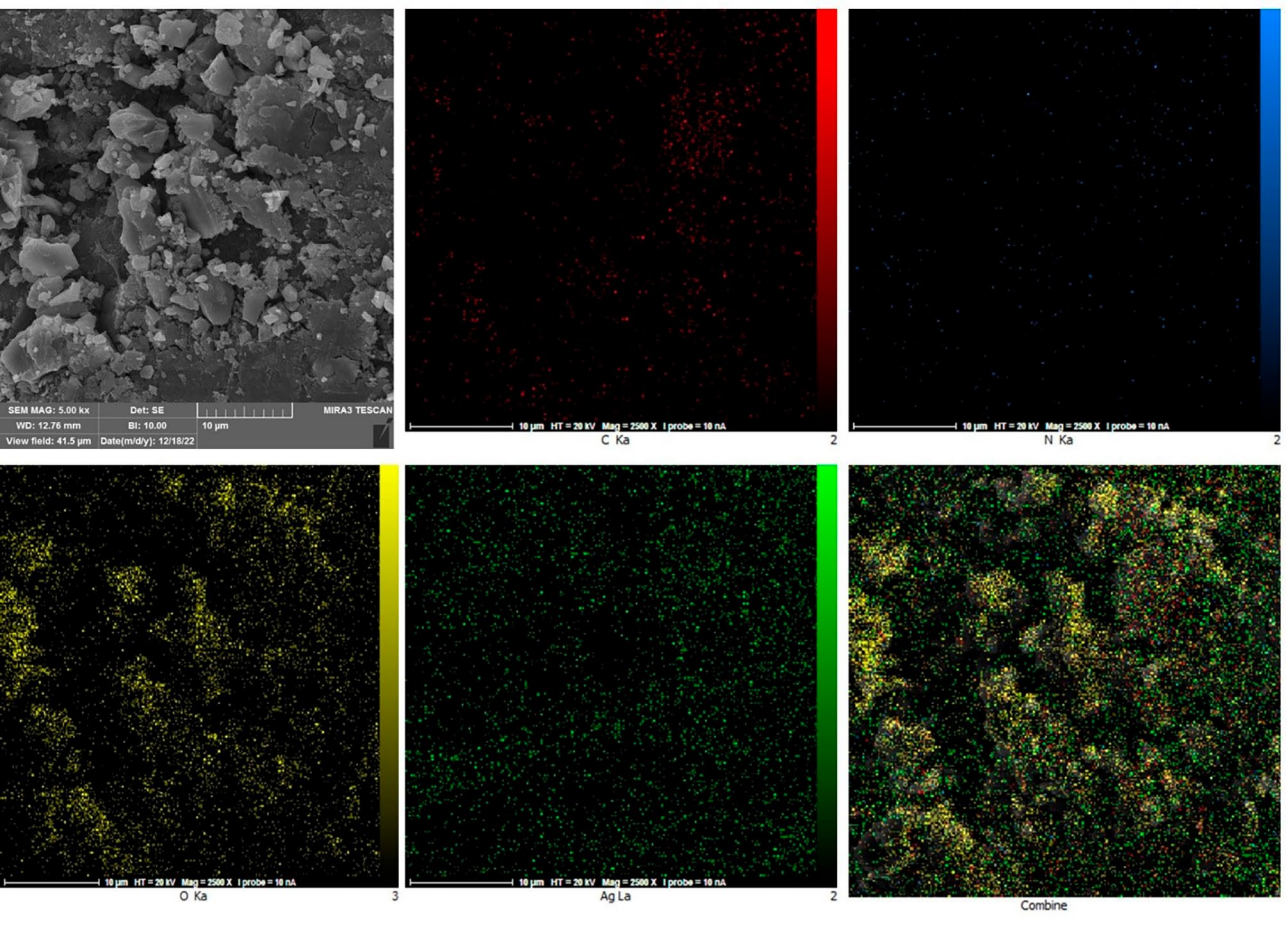
The distribution map of nanocomposite elements evaluated by SEM-EDX

The distribution map of nanocomposite elements is shown in Fig. 4. This figure shows that silver nanoparticles are well-distributed and completely non-agglomerated on the surface of chitosan nanocomposite. An essential point in the synthesis of multi-element nanostructures is the non-agglomeration of nanoparticles.

### Statistical analysis

The non-parametric Kolmogorov-Smirnov test indicated that the frequency distribution of the number of grown colonies in groups 1 to 3 followed a normal distribution (*P* = 0.200). In contrast the distribution in groups 4 and 5 was non-normal (*P* < 0.001).

Moreover, the assumption of homogeneity of variance of the number of grown colonies in the studied groups was also evaluated. Levene’s test showed that the assumption of homogeneity of variance was not met in the studied groups (*P* < 0.001). After performing the non-parametric Kruskal-Wallis H test (presented in Table 1), it was found that the median number of grown colonies varies significantly between the studied groups (*P* < 0.001). Therefore, it is necessary to compare the median number of grown colonies in “pairs” of the examined groups to determine which groups have a significantly higher median number of grown colonies. To this end, the non-parametric Mann-Whitney U test with Bonferroni correction was used.

**Table 1:** Data are reported as median (first quartile- third quartile) ^*^Kruskal-Wallis nonparametric test was statistically significant *P* < 0.05

Group Variable	(n = 6) 1	(n = 6) 2	(n = 6) 3	(n = 6) 4	(n = 6) 5	*P* Value^*^
The number of colonies	32/50 (29/00–37/75)	(17/75–23/25)20/00	(4/50–10/25)6/50	(0–0/25) 0	(0–0/50) 0	< 0/001

The result of the non-parametric Mann-Whitney U test for comparing the median number of grown colonies in pairs of the studied groups showed that:

- There is no significant difference in the median number of grown colonies between groups 4 and 5 (U = 500.17, *P* = 0.937).
- There is a significant difference in the median number of grown colonies among the other groups (U = 0.000, *P* < 0.002).
- In conclusion, the median number of grown colonies in Group 1 is significantly higher than in Group 2, while the median number of grown colonies in Group 2 is significantly higher than in Group 3. Similarly, the median number of grown colonies in Group 3 is significantly higher than in Groups 4 and 5. However, no significant difference in the median number of grown colonies was observed between Groups 4 and 5.

## Discussion

Tissue conditioners are temporary soft linings applied on the base of the resin prosthesis. They are used to reduce the complications of denture stomatitis in elderly patients, many of whom have limited mobility, cognitive impairment, and reduced memory, and reduce the need for dental visits and require minimal effort from the patient. However, patients with denture stomatitis often show poor prosthetic hygiene and use dentures for a long time, as a result, low pH and relatively anaerobic conditions often arise between the base of a denture or tissue conditioner and lining mucosa. This acidic environment allows C. albicans to grow and enhances the enzyme activity of proteinase and lipase. Studies have shown that the surface of tissue conditioner can accommodate C. albicans and intensify denture stomatitis [21]. Combining antifungal agents in tissue conditioners creates an effective antimicrobial agent barrier between the site of infection and healthy tissues.

In a study, Taheri et al. showed that chitosan has high antifungal properties and showed a significant difference from the usual antifungal drugs (including streptomycin, gentamicin, tetracycline, and erythromycin) [22]. Also, the in vitro study of Ballal et al. showed that the effect of chitosan on the growth inhibition of C. albicans is more than chlorhexidine, and the combination of 2% chlorhexidine gel with 2% chitosan gel has the maximum growth inhibition on C. albicans [23]. In the present study, the antifungal property of Nystatin, was compared with silver chitosan nanoparticles, which showed a much higher antifungal effect of silver chitosan on C. albicans.

In a study, Mousavi et al. showed complete inhibition of growth in 24 and 48 h for C. albicans, at a concentration of 2.5% and for Enterococcus faecalis, Pseudomonas aerogenes, and Streptococcus mutans and at a concentration of 5% Ag nanoparticles, ZnO, and chitosan [24]. In the present study, the complete inhibition of the growth of C. albicans colonies was done in 24 h at 2 MIC or equivalent to 0.19 mg/ml of chitosan along with green synthesized silver nanoparticles. The MIC of chitosan and green synthetic silver nanoparticles was 0.097 mg/mL. Therefore, indicating a better antifungal activity of these particles compared to Mousavi’s study. These findings can be related to the ability of low molecular weight chitosan to interfere with metabolic activity, disrupt the cell wall and prevent the adhesion of C. albicans and the synergistic effect of green synthesized silver particles. Green synthesized silver nanoparticles have antifungal and antimicrobial activity against gram-positive and gram-negative bacteria. The green synthesis of nanoparticles using plant extracts can help increase their antimicrobial properties significantly [25]. In the present research, it was shown that the extract of the Pistacia plant containing silver nanoparticles has a noticeable synergistic effect on the antifungal properties of chitosan nanoparticles.

The contemporary problem with using Nystatin and other standard antifungal agents is the development of cross-resistance against some Candida species and, more commonly, tissue irritation, digestive symptoms, and skin rashes. The proposed mechanism for the distinct antifungal behavior of chitosan involves the formation of a permeable chitosan film on the surface of the cell wall, which interferes with fungal growth and also activates several defense reactions which leads to inhibition of DNA/RNA synthesis and disruption of protein synthesis [25].

The mean number of colonies in the nystatin group decreased compared to the control and this decrease was clinically significant. When the number of colonies of all chitosan silver and nystatin groups was compared, the difference was statistically significant, although both chitosan silver and nystatin groups relatively inhibited biofilm formation.

The number of colonies in 1 MIC was compared with the 2 and 4 MIC silver chitosan group, the difference was statistically significant. However, growth inhibition between 2 and 4 MIC groups was not statistically significant and it seems that the 2 MIC concentrations and above have a relatively full inhibitory effect on the growth of Candida. As a result, the use of concentrations above 2 MIC does not increase the antifungal property; it also increases the manufacturing cost, darkens the material, and reduces the acceptability for the patient to use synthesized material.

Aksungur et al. investigated the in-vitro and in-vivo effects of chitosan and nystatin to treat chemotherapy-induced oral mucositis. They observed that mucositis scores in the nystatin-treated groups were significantly lower in gel and suspension formulations than in those treated with chitosan gel alone [26] In the study by Ibrahim et al., which aimed to evaluate the gastroprotective effect of Ag NPs against ethanol-induced gastric ulcers in rats, a decline in the ulcer index and an increase in the percentage of ulcer prevention were observed [27]. In contrast, the current study assessed the inhibitory effect of silver-chitosan nanoparticles on denture stomatitis, which is caused by the growth of C. albicans. The results indicated that these nanoparticles were more effective than nystatin application. The varying outcomes of these studies could be attributed to differences in the diseases (mucositis and denture stomatitis) and their causes, or to variations between in vitro and in vivo conditions.

## Conclusions

In conclusion, we found that the combination of chitosan with green-synthesized silver nanoparticles, even in very low concentrations, significantly reduced the number of Candida cells adhering to the surface of the tissue conditioner. Furthermore, in terms of antifungal efficacy, this combination proved to be more effective than either chitosan or silver alone, as well as the conventional antifungal medication nystatin. Therefore, it could serve as a suitable substitute.

The limitation of this experiment was the use of a high load of Candida disproportionately to the natural environment of the mouth, as well as the lack of attention to the presence of other microorganisms in the development of denture stomatitis, which was not addressed in the present study, and it is suggested to pay attention to it in future studies.

Chitosan silver nanoparticles prepared in the study also had a dark color, which affected the color of tissue conditioner samples. It is recommended to conduct further investigations to modify the color of the prepared antifungal agent.

New research has shown promising results, indicating that it may be a viable approach to expanding the use of this approach to patients who wear dentures and especialy those who receive radiotherapy and chemotherapy. This provides an opportunity for further study of its key features in the field of public oral health.

### Electronic supplementary material

Below is the link to the electronic supplementary material.

Supplementary Material 1

## Data Availability

All data generated or analyzed during this study are included in this published article and its supplementary information files.

## Declarations

### Research Guidelines

We confirm that all experimental research and field studies involving plants, both cultivated and wild, presented in this submission have been conducted in accordance with relevant institutional, national, and international guidelines and legislation. The collection of plant material and the entire research process have followed established standards to ensure the ethical and responsible conduct of our study.
